# The Impact of Physical Activity on Sleep in Alcohol Users: A Systematic Review

**DOI:** 10.1111/adb.70050

**Published:** 2025-07-07

**Authors:** Lilou Duquet, Silvio Galli, Emmanuel Haffen, Julie Giustiniani

**Affiliations:** ^1^ Université Marie et Louis Pasteur, INSERM, UMR 1322 LINC Besançon France; ^2^ Sleep Exploration Department University Hospital Besançon France; ^3^ Psychiatry and Addictology Department University Hospital Besançon France

**Keywords:** alcohol, addiction, physical activity, sleep

## Abstract

Alcohol misuse impairs sleep quality and circadian rhythms. Yet, sleep is essential, as a lack of sleep is a predictive factor for addiction and relapse risk in patients with alcohol use disorder (AUD). On the contrary, effective insomnia treatment after withdrawal increases abstinence. Meanwhile, physical activity (PA) has been shown to improve sleep quality and circadian rhythms in nonclinical population. Hence, it would be interesting to assess the impact of PA on sleep in alcohol users with and without dependence. Systematic search was conducted using Prisma guidelines for the screening and ROB‐1 for bias analysis of randomized controlled trial (RCT). Out of 4995 studies screened, none assess as main purpose the impact of PA on sleep in alcohol users. Still, 81.8% of the selected studies, in their secondary outcomes, highlight PA's positive association with sleep in alcohol users with or without dependence. Main positive sleep outcomes were insomnia and sleep fragmentation reduction as well as sleep quality and duration improvement. There is a lack of publication regarding the impact of PA on sleep in nonclinical alcohol users and AUD patients. Still, PA appears to enhance sleep in both populations. Further well‐designed RCTs are needed to produce robust data. In the first instance, feasibility study should be performed as adhesion can be an issue in the population. Finally, different PA programs (frequency, intensity, time, type and duration) should be compared to determine the optimal dose in different AUD status (intoxication, withdrawal and abstinence).

## Graphical abstract

Alcohol misuse impairs sleep whereas physical activity enhances it in ordinary population. Furthermore, sleep disturbances increase consumptions and relapse risk in patients with alcohol dependence. Thus, exercise could be used as a nonpharmaceutic tool to improve sleep in alcohol users. Systematic search of the literature was performed to ensure exercise has been shown to enhance sleep in this population. 4995 articles arose, yet 11 were selected as others did not answer the problematic. None of the selected studies had as main purpose to assess exercise impact on sleep in alcohol users. Yet, in secondary outcomes, 81.8% highlight physical activity's positive association with sleep in alcohol users with or without dependence. More studies are needed to precise programs modalities according to patients’ alcohol use and sleep profile.

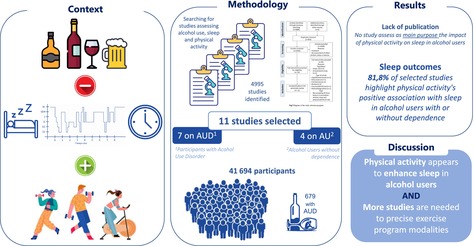

## 1 Introduction

Alcohol use disorder (AUD) causes 3 million deaths per year according to the World Health Organization (WHO). It is a public health issue worldwide with substantial risk of morbidity, affecting psychological, physical, social and professional areas [1]. This chronic disease is closely related to sleep disorder. Sleep disturbances, such as insomnia, are overrepresented in alcohol users with or without AUD [2]. Meanwhile, alcohol misuse impairs circadian rhythms and sleep quality while inducing subsequent insomnia symptoms [3].

### 1.1 Alcohol's Impact on Sleep

Sedative effect of acute drinking during the decline of blood alcohol induces sleep onset latency (SOL) reduction [4], which contributed to patients self‐medicating their sleep disturbances with alcohol [5]. Indeed, more than one out of 10 individuals use alcohol as a hypnotic agent to self‐medicate sleep problems [6]. Yet, subjective analysis revealed that AUD patients experiment insomnia symptoms, frequent nightmares [7] and 79% have poor sleep quality according to Pittsburg Sleep Quality Index (PSQI) [8]. These subjective results are confirmed by objective analysis [7]. Actigraphy analyses showed 75,89% sleep efficiency (SE) in AUDs, correlated with subjective self‐reported SE [8]. While subjective assessments provide valuable insights into an individual's perception of their sleep quality [7], both objective and subjective approaches may not always align [8, 9]. Reviews, combining subjective and objective tools, showed that *acute drinking* induces, in the 2nd part of the night, total sleep time (TST) and SE reduction, whereas multiple awakenings occur [2]. *Chronic drinking* induces downregulation of Glutamate transporter 1 (GLT‐1) [10] leading to excessive glutamatergic activity, disrupting sleep–wake cycles [11]. It increases sleep fragmentation, rapid eye movement (REM) sleep and reduces slow wave sleep (SWS) [2, 4]. Chronic drinking is also associated with circadian misalignment due to melatonin inhibition and reduced circadian photoreceptor responsivity [12, 13]. During *withdrawal*, AUD patients experiment SOL delay or augmentation as melatonin rise time and peak are delayed. Over time, recovery can occur, however, some changes may be resistant to restoration [4] such as increased REM sleep, sleep fragmentation, wake after sleep onset (WASO), SOL, stage 1 sleep, insomnia and reduced SWS, SE and TST [2, 6]. Thus, alcohol deteriorates sleep while a lack of sleep is a predictive factor for chronic diseases such as addiction.

### 1.2 Sleep's Impact on Alcohol Consumption

Sleep disturbances predict subsequent alcohol consumption and increase relapse risk in withdrawn patients [2, 14]. Precisely, sleep fragmentation and REM sleep augmentation as well as periodic leg movements are significant relapse predictors [6]. Use of alcohol as a sleep aid is also associated with relapse. Thus, sleep disturbances negatively interfere with addictive care and withdrawal. On the contrary, effective treatment for sleep disturbances aids recovery and increases abstinence [4, 6], highlighting the necessity of addressing sleep issues in AUD treatment programs. Furthermore, in this population, nonpharmaceutical interventions should be emphasized, such as adapted physical activity (APA), to avoid substance dependence transfer.

### 1.3 Exercise and Sleep

The positive effect of physical activity (PA) on sleep in the nonclinical population has been proven. Indeed, a*cute exercise—*defined as less than 1 week of aerobic and/or anaerobic exercise—has a small positive effect on TST, SOL, SE, SWS—augmentation and REM sleep—reduction, and small‐to‐medium effect on WASO and stage 1 sleep—reduction, if performed at least 3 h before bedtime [15]. These immediate benefits provide the right contingency to motivate exercising regularly to improve sleep [15]. *Regular exercise*—defined as equal or greater than 1 week of aerobic and/or anaerobic exercise, in that case 10‐ to 16‐weeks programs—showed small benefits on TST and SE and small‐to‐medium positive effect on SOL. It also has moderate‐to‐large benefits on all PSQI subscales except sleep medication. Yet, a study showed that exercise reduces the use of medication to assist sleeping [16]. Large positive effect on overall sleep quality (PSQI) especially on those with initial sleep complaints was found [15, 16, 17]. PA also regulates circadian clock network synchronization; it can both reduce and increase melatonin (MT) concentration according to the time of day, intensity of light, intensity and duration [12]. Regular exercise also decreases cortisol level, which is correlated to TST evolution [18]. Furthermore, PA reduces total wakefulness and increases sleep depth and performance. Finally, in insomnia patients, exercise decreases sleep anxiety for up to 5 h [17].

While direct studies investigating mechanisms underlying PA's positive impact on sleep in individuals with AUD are limited, existing research provides insights into potential mechanisms. A deficit of GLT‐1 is a common neuromodulation observed in chronic AU, inducing hyper‐glutamatergic activity responsible for sleep structure imbalance [10, 11]. PA increases GLT‐1 improving glutamate reuptake [19]. Therefore, it could prevent sleep structure disruption in chronic AU by regulating glutamatergic activity. The impact of PA on sleep is a multifactorial system, and many other mechanisms, described in the cited meta‐analysis, may be involved, including other neuromodulations, circadian rhythms regulation, fatigue, cytokine evolution, anxiety reduction, temperature fluctuation and so on [15].

There is no general agreement regarding the procedure to follow in terms of PA to enhance sleep in nonclinical population. Indeed, the effects of frequency, intensity, time, type, duration (FITT‐D) are unclear yet and may not moderate its positive effects on sleep [15].

The literature is well established on the links between sleep and alcohol and on the benefits of exercise on sleep and alcohol use separately [20]. It seems this nonpharmaceutical tool would be particularly suitable to prevent or treat sleep disturbances in Alcohol Users ‘AU’, defined as nonclinical people drinking alcohol without diagnosed dependence, and in ‘AUD’, patients defined as those diagnosed with alcohol dependence [21]. Nevertheless, very little is known on the efficiency of exercise as a therapeutic to improve sleep quality in this population. In that regard, the purpose of this review is to establish the state of knowledge by gathering all the scientific studies published, assessing the impact of exercise on sleep in AU and AUD. Another goal is to submit suggestions for researchers on how to measure, with well‐designed trials, PA's impact on sleep outcomes in this population.

## 2 Method

### 2.1 Eligibility Criteria

Studies were eligible if they were prospective randomized or nonrandomized, published in English, French or Spanish and evaluated the impact of PA on sleep in AU or AUD. The inclusion criteria were studies involving human participants (18 years and older), with any types of alcohol use. Studies were considered eligible if they reported PA habits or included exercise interventions. The intervention could be supervised and/or home‐based, whatever the FITT‐D of exercises. To be included, trials had to evaluate sleep outcomes as primary or secondary outcome using self‐reported or objective assessments.

### 2.2 Information Sources and Search

**FIGURE 1:**
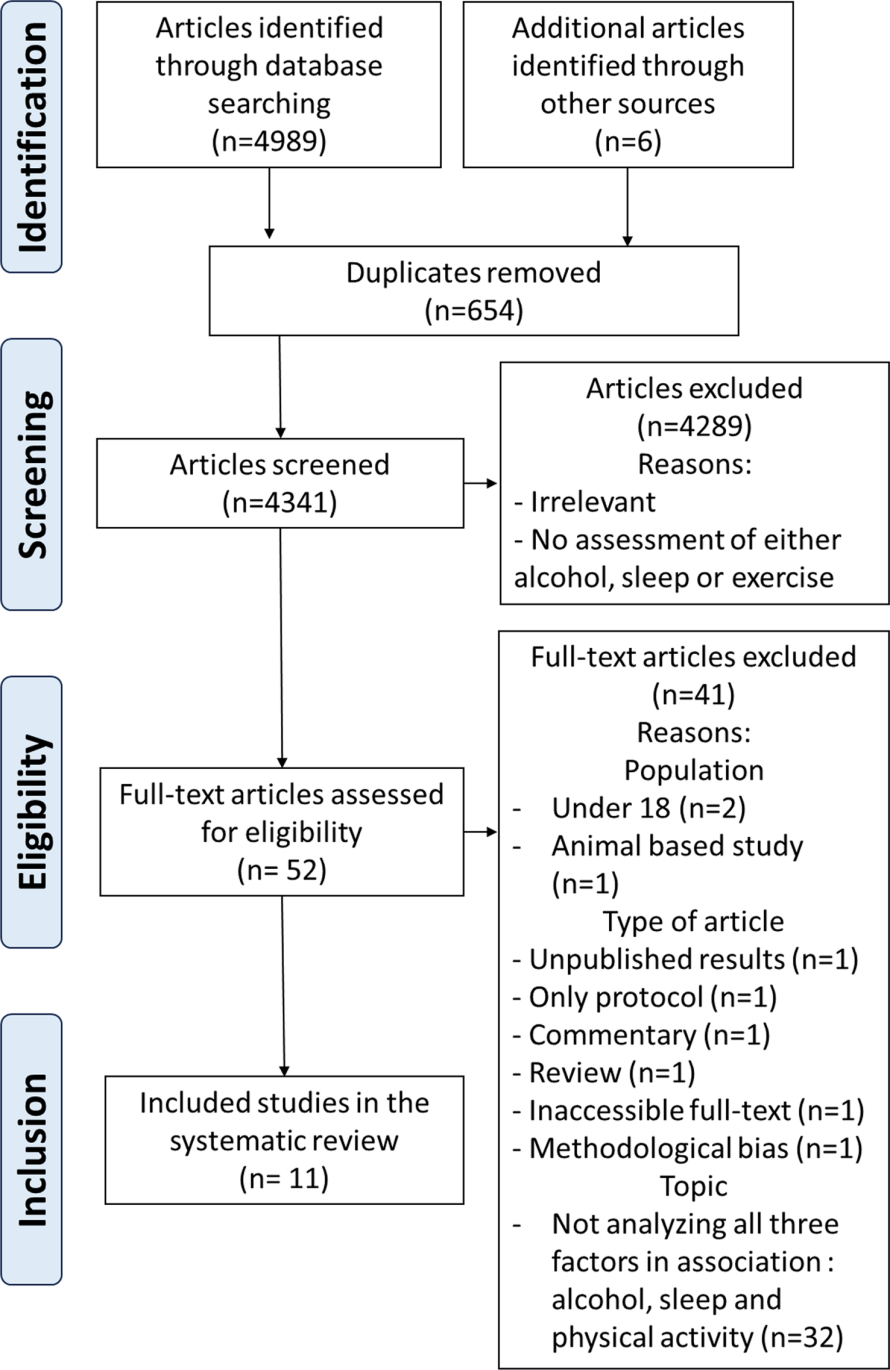
Diagram of the study selection program.

Eligible studies were identified by a systematic search in Embase, Central and PubMed bibliographic databases, conducted from December 2023 to January 2024 (Figure 1). In total, two team members independently screened studies for inclusion, extracted data and assessed the risk of bias (Figure 1). The data were presented as a narrative synthesis. Participants' characteristics, study design, exercise interventions and results were selected following the PICO (population, intervention, comparator and outcomes) as follows: (1) P (population), alcohol related disorder, alcohol; (2) I (intervention), exercise, PA*, gymnastics*, aerobic exercise, pilates, bicycling, physical conditioning, endurance training, resistance training, running, jogging, sport*, exercise therapy, swimming, walking, nordic walking, dancing, dance therapy, cycling, exercise movement techniques, rehabilitation, physical education and training; (3) C (comparator), all other interventions; and (4) O (outcomes), sleep*, sleep wake disorders, insomnia*. The search was conducted using a combination of Medical Subject Headings (MeSH) and was adjusted to the characteristics of each database. Each search term was linked with ‘OR’ and ‘AND’ to search for relevant literature. The Preferred Reporting Items for Systematic Reviews and Meta‐Analyses (PRISMA) protocol was applied [22].

### 2.3 Data‐Collection Process

Once the search was done and after duplicates were removed, the titles and abstracts of identified studies were examined independently by two researchers to identify studies meeting the selection criteria. Then, the full text of selected studies was assessed by two investigators. Several were excluded due to population, topic and/or type of article not meeting selection criteria or unpublished results. Methodological quality of the four randomized controlled trials (RCTs) was examined using the risk of bias criteria recommended by the Cochrane Collaboration: Risk of Bias 1.0 tool (RoB1) [23]. Two independent reviewers also carried out the scoring.

## 3 Results

### 3.1 Study Selection

A total of 4995 records, published in Embase (*n* = 2195), PubMed (*n* = 2691), Central (*n* = 103) and through other sources (*n* = 6), were identified. After the removal of 654 duplicates, 4341 were initially screened. Of these 4341 papers, 4289 were excluded based on our exclusion criteria (Figure 1). The full text of the remaining 52 articles was retrieved and reviewed. Forty‐one of these were subsequently excluded because they did not meet selection criteria regarding population, such as age, type of article (reviews and protocols) or topic; indeed, most articles did not analyse exercise, sleep and alcohol in association. A final total of 11 studies were included in the review.

### 3.2 Study Characteristics

This review includes 11 human‐based studies, gathering a total of 41 694 participants, 229 in interventional and 41 465 in longitudinal studies. Seven studies are based on patients with AUD, where two include also Substance Use Disorder (SUD) patients, which make a total of 679 AUD and 19 SUD patients. Four remaining longitudinal studies are based on 40 996 AU. All studies present data offering information on the impact of PA on sleep in alcohol users with or without AUD. They either compare exercise group (EX) to usual care group (UC) or to patients with various PA levels or sleep disturbances. Characteristics of the studies and participants are summarized in Table 1 for interventional studies and in Table 2 for others. Table 3 diplays studies sectioned by type of research and population.

**TABLE 1:** Interventional studies characteristics.

Reference	Sample*N* = totalEX^a^ *n* = xxUC^b^ *n* = xxM = global mean age or EX and UC Gender (m:f)	Study designType of study Participant's environment	Population characteristics Addiction *Severity* *Historic* *Consumption: units per week* *Withdrawal* Sleep Physical activity	Usual care	Exercise intervention
AUD^c^					
Gary et al. 1972 [24]	*N* = 20 UC + EX *n* = 10 UC *n* = 10 M = 38.9 (20:0)	RCT^d^ Inpatient alcoholic treatment ward	**Addiction** AUD Severity: NI Historic: 18 years of problem drinking Consumption: NI^e^ Withdrawal: NI **Sleep** NI **PA** ^f^ Time for exercise pulse rate to return to standing: 139.5 s	Group psychotherapy, ward clean‐up and recreation programs Medication: NI	4 weeks Jogging a mile‐course 5 days per week until a total of 20 mile has been reached.
Ermalinski et al. 1997 [25]	*N* = 90 UC + EX *n* = 48 UC *n* = 42 M = 41.6 and 39,4 (90:0)	RCT Inpatient alcohol rehabilitation	**Addiction** AUD Severity: NI Historic: Mean number of previous hospitalization UC 1.2; EX 9 Consumption: NI Withdrawal: NI **Sleep** NI **PA** NI	Psychotherapeutic group with psychologist, psychotherapy, didactic sessions and incentive therapy Medication: NI	6 weeks 10‐min 5 days a week of BMC^g^: abdominal breathing, yoga stretches ‘Sun salutation’ + Jogging 5 min per day adding 1 min each day until reaching 20.
Hallgren et al. 2014 [26]	*N* = 14 UC + yoga *n* = 6 UC *n* = 8 M = NI NI	RCT Outpatient alcohol treatment clinic	**Addiction** AUD Severity: AUDIT^h^ UC 17.33; EX 20.36 Historic: CDT^i^ UC 0.37; EX 1.15 Consumption: UC 17.66; EX 35.56 Withdrawal: no **Sleep** NI **PA** NI	Psychotherapy Medication: Treatment for alcohol dependence	10 weeks Yoga intervention, 1 session of 90 min per week combining light PA, meditation and deep breathing techniques. Encouraged to practice once a day autonomously.
SUD including AUD					
Flemmen et al. 2014 [27]	*N* = 16 UC + HIIT^j^ *n* = 9 UC *n* = 7 M = 33 and 31 (13:3)	RCT Inpatient long‐term treatment (3 months)	**Addiction** SUD^k^ 2 in HIIT group had AUD AUD severity: NI Historic: UC 12 years of abuse EX 17 Consumption: NI Withdrawal: NI **Sleep** NI **PA** No endurance training for the last 6 months	Ballgames, yoga, stretching, out‐door walking, low resistance, strength training, ceramics, card games, TV games (< 70% HRmax^l^) Medication: 2 had substitutional treatment	8 weeks UC + HIIT: supervised training 3/week Inclined walking or running on treadmill. HIIT: 4 × 4 min of aerobic intensity (90%–95% HRmax) interrupted by 3 min recovery periods (70% HRmax)
Berger et al. 2023 [28]	*N* = 89 PA level groups Low *n* = 51 Moderate *n* = 21 High *n* = 17 M = 54 (68:21)	Original research Inpatient long‐term treatment (> 30 days)	**Addiction** SUD 87,64% with AUD AUD severity: SDS^m^ high 9; low and moderate 10 Historic: GGT^n^ low 46; moderate 41; high 25 Consumption: NI Withdrawal: NI **Sleep** % of participants with insomnia: low 63; moderate 30; high 35 **PA** NI	Group and/or individual psychotherapy sessions and help with social challenges No exercise intervention Medication: treatment for addiction	

^a^ Usual Care.

^b^ Exercise.

^c^ Alcohol Use Disorder.

^d^ Randomized Controlled Trial.

^e^ No Information.

^f^ Physical Activity.

^g^ Body Mind Component.

^h^ Alcohol Use Disorder Identification Test.

^i^ Carboxy Deficient Transferine.

^j^ High Intensity Interval Training.

^k^ Substance Use Disorder.

^l^ Heart Rate maximal.

^m^ Severity Dependence Scale.

^n^ Gamma Glutamyl Transferase.

**TABLE 2:** Longitudinal and cross‐sectional studies characteristics.

Reference	Sample *N* = Total Group 1, *n* = xx Group 2, *n* = xx M = mean age Gender (m:f)	Study design	Population	Alcohol use M = mean alcohol units per week *Severity* *Historic: years of abuse* *Consumption: units per week* *Withdrawal*	SleepM = mean hours per night at baseline	Physical activityM = mean PA in minutes per week at baseline
AUD^a^						
Bolstad et al. 2023 [29]	*N* = 87 Insomnia *n* = 47 Control *n* = 40 M = 54.1 and 54.3 (65:22)	Longitudinal study	Patients with AUD inpatient long‐term treatment for SUD^b^ (1 to 9 months) Medication: medication for somatic and psychiatric diagnosis. Probable sleep aid medication	AUD Severity: SDS^c^ score 10 Historic: 15 to 16 Consumption: NI^d^ 15% to 20% had other SUD Withdrawal: 17 to 20 days since last drink	54% insomnia	40,23% PA^e^ level > moderate
Ternay et al. 2022 [30]	*N* = 382 PA level groups Insufficient *n* = 103 Sufficient *n* = 279 M = 38 (266:116)	Cross‐sectional study	Patients with AUD endorsed in outpatient for addiction consultation Medication: NI	AUD Severity: DSM‐5^f^ score 7; AUDIT^g^ 23 Historic: NI Consumption: NI 13.3% concurrent opioid use 33.0% concurrent stimulant use Withdrawal: NI	Median PSQI^h^ score: 9 (> 6 = sleep disturbances)	26.18% insufficient PA level
AU						
Tracy et al. 2021 [31]	*N* = 70 M = 26.71 (27:43)	Cross‐sectional study	Nonclinical population with ‘normal’ or ‘late’ sleep timing Medication: no medication affecting sleep, melatonin, metabolism or for psychiatric disorder	AU Severity: NI Historic: NI Consumption: M = 3.96 22.1%: 0 units per week 57.3%: 1 to 7 17.7%: 7 to 14 2.9%: > 14	M = 7.25	M = 151.47
Hajat et al. 2019 [32]	*N* = 34 061 PA level groups Low *n* = 13 760 Moderate *n* = 8248 High *n* = 12 053 M = 42 (14881:19180)	Longitudinal study Mean duration	Nonclinical population Medication: NI	AU AU severity: NI Historic: NI Consumption: M = 2.4 Low 2.3; moderate 2.6; high 2.5	M = 6,9	M= High: 282.9 Moderate: 98 Low: 62.8
Mudryj et al. 2019 [33]	*N* = 6789 M= > 18 (3360:3429)	Cross‐sectional study	Nonclinical population Treatment: NI	AU Severity: 26.6% high‐risk alcohol use Historic: NI Consumption: high risk = > 5 units per occasion > 2 times/month OR > 1 unit/day every day for a year	36% inadequate sleep	NI
Bosch et al. 2017 [34]	*N* = 76 M = 36.42 (62:14)	Longitudinal study	Veterans with PTSD^i^ Treatment: NI	AU Severity: MAST 4.54 Historic: NI Consumption: NI	NI	NI

^a^ Alcohol Use Disorder.

^b^ Substance Use Disorder.

^c^ Severity Dependence Scale.

^d^ No Information.

^e^ Physical Activity.

^f^ Diagnostic and Statistical Manual of Mental Disorders 5.

^g^ Alcohol Use Disorder Identification Test.

^h^ Pittsburg Sleep Quality Index.

^i^ Post‐Traumatic Stress Disorder.

**TABLE 3:** Studies sectioned by alcohol use characteristics and type of article.

	Interventional studies	Longitudinal and cross‐sectional studies
**AUD**	Gary et al. 1972	Bolstad et al. 2023
	Ermalinski et al. 1997	Ternay et al. 2022
	Hallgren et al. 2014	
**SUD including AUD**	Flemmen et al. 2014	
	Berger et al. 2023	
**Nonclinical alcohol use—AU**		Tracy et al. 2021
		Hajat et al. 2019
		Mudryj et al. 2019
		Bosch et al. 2017

In the seven studies based on AUD, the sample ranges from 14 to 382 gathering a total of 679 participants. Average age ranges from 31 to 54.3 years and women representation ranges from 0% to 30.4%. Four of these studies are RCT [24, 25, 26, 27]; two sample the subject by PA level [28, 30] and one according to insomnia [29]. This last is the only study indicating clearly that participants were abstinent [29]. One RCT clearly shows that participants are still active consumers [26]. In all others, withdrawal was not clearly specified nor consumptions; hence, it is unclear whether participants were abstinent or not. Two studies include patients with SUD [27, 28]; in one case, 87,64% have AUD [28], and in the other, two out of 16 [27].

Concerning PA, all studies seem to assess regular exercise. RCT assess programs lasting 4 to 10 weeks [24, 25, 26, 27]. Participants did two to five sessions per week of 10 to 90 min. The intensity of the exercise was specified in only one study, using high intensity interval training (HIIT) [27]. Other studies seem to assess light to moderate exercise programs [24, 25, 26]. Finally, concerning type of exercise, one study did not specify [28], two did aerobic protocols (running, walking) [24, 27], one had mind–body activities (yoga) [26] and the last one combined both aerobic and anaerobic exercise [25].

For the four studies based on AU, the sample ranges from 70 to 34 061 gathering a total of 40 996 participants. Average age ranges from 26.7 to 42 years and women representation ranges from 18.4% to 61.4%. Three are based on nonclinical population [31, 32, 33], and the last one on veterans with post‐traumatic syndrome (PTSD) [34]. Two studies showcase alcohol consumption with means of 2.4 and 3.96 units per week [31, 32].

### 3.3 Methodological Quality of the RCT

**FIGURE 2:**
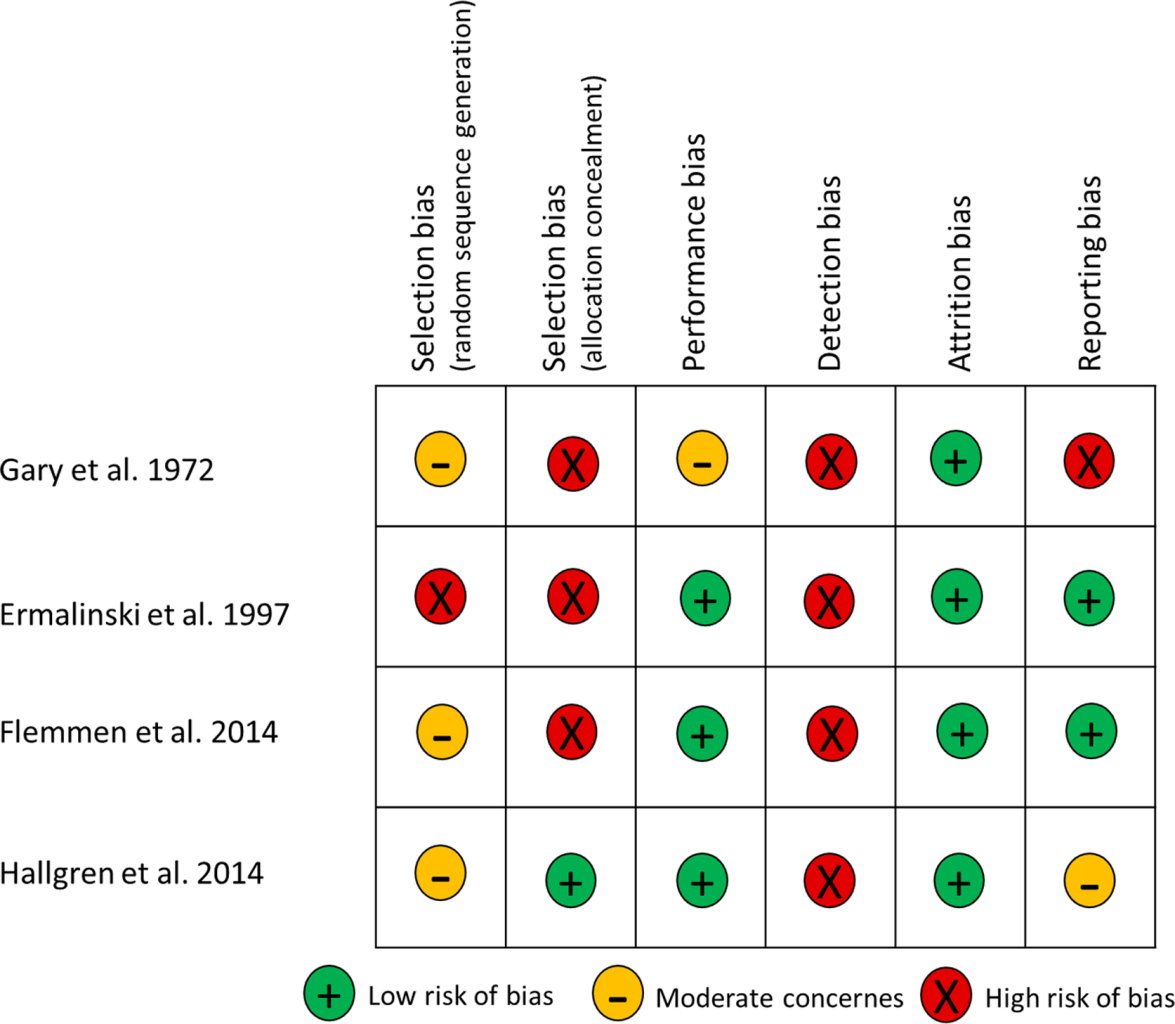
Risk of bias in randomized controlled trial studies.

The risk of bias for RCTs is summarized in Figure 2. Regarding performance and attrition, all studies have low risk of bias, except Gary et al., which has insufficient information for performance bias, inducing moderate concerns. All RCTs have insufficient information for random sequence generation bias elevating moderate concerns except Ermalinski et al., in which high risk was identified. Hallgren et al.'s study has low risk of allocation concealment; nevertheless, all other RCTs show high risk. Meanwhile, all RCTs had high risk detection bias since there were no information in any articles, and the participants could not be blinded in all of them due to study design. Finally, reporting bias is high in Gary et al.'s study; since no table showed results regarding our topic of interest, it was only described in the text. There are moderate concerns in Hallgren et al., meanwhile, the other RCTs have low risk of reporting bias.

### 3.4 Effect of Exercise on Sleep in Alcohol Users With or Without AUD

Main results are displayed in Table 4, which is organized in two main sections: AUD and nonclinical AU. It also presents main purpose, adherence rates and type of analysis.

**TABLE 4:** Main results of studies assessing the impact of exercise on sleep in alcohol users with and without diagnosed disorder.

Reference	Main purpose	Adherence	Analysis			Main results
			Sleep	Alcohol	PA^a^	
			Timing of analysis Measuring tools			
**AUD** ^b^						
Gary et al. 1972 [24]	Effects of jogging on physical fitness and self‐concept in AUD	Dropout 55.55% Conflicting activities, job, ECG^c^ results	Logs maintained for 4 weeks		Baseline + Week 4	EX^d^ experienced lower level of sleep disturbances.
			Logs	Logs	Cardiovascular	
Ermalinski et al. 1997 [25]	Impact of body mind treatment on alcoholic inpatients	NI^e^	Twice a week for 6 weeks		Baseline + Week 6	Neither group of AUD patients had significant changes in satisfaction with sleep even those who did exercise
			‘Satisfaction with sleep’ 9‐point scale	Cravings 9‐point scale	Cardiovascular, muscular capacity	
Hallgren et al. 2014 [26]	Impact of yoga as an adjunct treatment for alcohol dependence	77.7%	Baseline + Month 6		NI	Improvement in sleep duration and quality wa0073 described by patients from the yoga group during the semi‐structured interview.
			Interview	DSM‐4^f^, TLFB^g^		
Bolstad et al. 2023 [29]	Identify determinants of insomnia among patients with AUD	49% did not consent	Baseline + Week 6 + Month 6	Baseline + Week 6	Baseline + Week 6 + Month 6	Moderate or high PA level associated with lower risk of insomnia Low PA associated with insomnia at baseline and 6‐week follow‐up. % of participants with insomnia: Baseline 54%; Week 6 35%; Month 6 37%
			SCI^h^	PEth^i^, SDS^j^, ICD‐10^k^	IPAQ‐SF^l^	
Ternay et al. 2022 [30]	Identify insufficient PA as a global marker of severity in AUD patients	NI	Baseline			Insufficient PA group had deteriorated quality of sleep with higher PSQI scoring 11 against 9 for sufficient PA group
			PSQI^h^	DSM‐5^f^, AUDIT^m^, SADQ^n^	IPAQ^l^	
SUD^o^ including AUD						
Flemmen et al. 2014 [27]	Impact of HIIT^p^ in patients with SUD	EX: 75% vs. UC^q^: 58.3% Attended at least 83.3% of the sessions	Baseline + Week 8	NI	NI	ISI score reduction however not significant for either group. No significant difference between groups.
			ISI^r^			
Berger et al. 2023 [28]	Association between regular exercise and depressive symptoms among AUD and SUD	89%	Baseline			Level of PA was closely related to insomnia in AUD and SUD. % of patients with insomnia between groups: Low 63%; moderate 30%; high 35%
			SCI	y‐GT^s^, Peth, SDS	IPAQ‐SF	
**Nonclinical AU** ^**t**^						
Tracy et al. 2021 [31]	Assess relationship between sleep and PA and the moderating role of daily alcohol consumption	NI	Baseline			Longer daily total PA duration reduces sleep fragmentation on daily and average (7 day) level, in those with lower alcohol intakes (below mean = 3.96/week). Longer WASO^u^ associated with shorter PA duration in those with higher alcohol intakes (above mean)
			7‐days wrist actigraphy	7‐days log	Sense wear Pro Armband monitor	
Hajat et al. 2019 [32]	Association between PA and improvements in another lifestyle behaviour	NI	Baseline + reported during in average 40.1 months			In the year following PA initiation, participants showed an improvement in sleep (7.1 to 7.2 h/night).
			Self‐reported sleep duration	Self‐reported weekly units	Self‐reported weekly PA	
Mudryj et al. 2019 [33]	Relation between health‐related behaviours in Canadian adults	Response rate Cycle 3 (2012–2013) 51.7% Cycle 4 (2014–2015) 53.7%	Baseline			40.5% of those with inadequate PA (< 30 min on at least 5 days/week) have inadequate sleep (< 6 or > 10 h/day or > 2 sleep troubles).
			Self‐reported sleep duration and troubles	Self‐reported consumption in units	Activity monitoring device	
Bosch et al. 2017 [34]	Impact of exercise on sleep quality in veterans with PTSD^v^	NI	Baseline + 1‐year follow‐up			Engaging in exercise at baseline was associated with better sleep quality at 1‐year follow‐up
			PSQI	MAST^w^	Sessions per week + intensity	

^a^ Physical Activity.

^b^ Alcohol Use Disorder.

^c^ Electrocardiogram.

^d^ Exercise Group.

^e^ No Information.

^f^ Diagnostic and Statistical Manual of Mental Disorders 4 or 5.

^g^ Timeline Follow‐Back.

^h^ Sleep Condition Indicator.

^i^ Phosphotydilethanol.

^j^ Severity Dependence.

^k^ Scale International Classification of Disease 10.

^l^ International Physical Activity Questionnaire (Short form).

^m^ Alcohol Use Disorder Identification Test.

^n^ Severity of Alcohol Dependence Questionnaire.

^o^ Substance Use Disorder.

^p^ High Intensity Interval Training.

^q^ Usual Care group.

^r^ Insomnia Severity Index.

^s^ GammaGlutamylTranspeptidase.

^t^ Alcohol Users.

^u^ Wake After Sleep Onset.

^v^ Post‐Traumatic Stress Disorder.

^w^ Michigan Alcohol Screening Test.

#### 3.4.1 Assessments

All studies based on AUD assess sleep with subjective tools. Five with questionnaires and logs [24, 27, 28, 29, 30] and two with semi‐structured interviews [26] or question with a scale on satisfaction with sleep [25]. Regarding alcohol use, one uses a question with a scale on cravings [25]. Five others either use logs or questionnaires [24, 26, 28, 29, 30] and/or objective analysis with blood samples of alcohol use biomarkers [28, 29]. Concerning physical condition, three use International Physical Activity Questionnaire (IPAQ) [28, 29, 30], and two use objective markers of cardiovascular capacity [24, 25]. Adherence is given in five of the seven studies [24, 26, 27, 28, 29].

The only study assessing sleep objectively with actigraphy is based on AU [31]. Three other studies on AU use questionnaires [34] and self‐reported sleep duration [32, 33]. Regarding alcohol use, three studies present self‐reported consumptions in units [31, 32, 33] and one uses a questionnaire [34]. As for PA, two studies objectively monitor activity [31, 33], and two gather subjective information on weekly PA [32, 34]. Finally, only one study based on AU gave adherence rate.

#### 3.4.2 Main Results

First main result from this review is that, out of 4995 articles screened, none assess the impact of PA on sleep in alcohol users with or without AUD as their main purpose.

Second main result is that 81,8% of the selected studies indicate that PA significantly enhances sleep, or that the lack of it has a negative impact in both AU and AUD [24, 26, 28, 29, 31, 32] while none indicate that PA deteriorates sleep.

Concerning AUD, all sleep analysis was subjective. One study indicates that PA enhances sleep quality and duration [26]. Four studies showed that PA is closely related to sleep disturbances [24], such as insomnia [27, 28, 29]. Indeed, the level of PA is positively associated with insomnia in two studies [28, 29], with twice as many insomnia patients in low PA group compared to moderate [28]. Studies also showed insomnia reduction following PA augmentation going from 54% with insomnia at baseline to 35% at Week 6, with long‐term enhancement as rate stays at 37% at 6‐month follow‐up [29]. Another showed insomnia reduction however not significant [27]. Finally insufficient PA appears to deteriorate sleep quality as a two points different at PSQI between patients with sufficient and insufficient PA level was shown [30]. One study did not show any changes in satisfaction with sleep following a 6‐weeks PA program [25].

In AU, studies came to similar conclusions showing increased subjective sleep duration [32] and sustainable improvements in subjective sleep quality, lasting until 1‐year follow‐up [34]. A study also showed that longer PA in the day reduces objective sleep fragmentation that night, for those with lower consumptions than average (M = 3.96 units/week). Individuals with higher consumption did not receive the benefit [31]. Finally, 40,5% of AU doing inadequate PA—under 30 min PA at least 5 times a week—experienced inadequate sleep—either too short (< 6 h), too long (> 10 h) sleep duration and/or several sleep disturbances [33].

Regarding adherence, in AUD studies offering PA program showed 75% to 77.7% [26, 27] adherence rates and 55.55% dropout appeared [24]. Two other studies, not providing PA program, showed in one case an 89% adherence rate [28], and in the other, 49% did not give consent to participate [29]. In AU adherence is displayed in one study and ranges from 51.7% to 53.7% [33].

## 4 Discussion

### 4.1 Lack of Publication

Firstly, this review highlights a lack of publication regarding our questioning. Indeed, to date, no article whose main purpose is to assess PA's impact on sleep in AU or patients with AUD has been published. This is even more surprising given that negative association between sleep disorders and alcohol consumptions has been proven as well as exercise's positive impact on sleep and AUD. Thus, now, researchers should focus on studying the impact of PA on sleep in AU and especially patients with AUD.

### 4.2 The Impact of PA on Sleep in AU and AUD

Even though it is not the study's main purpose, the analyses of secondary outcomes still showed promising results regarding the positive impact of PA on sleep in both AU and AUD.

#### 4.2.1 Sleep Outcomes Subjectively Measured

In AUD, the selected studies identify PA as a key factor to reduce perception of sleep disturbances [24] such as insomnia, as they present subjective insomnia reduction in those with sufficient compared to insufficient PA level [28, 29]. In AU, inadequate PA also induces subjective inadequate sleep [33]. These results are consistent with recent meta‐analysis, based on patients with insomnia, showing that PA has a positive effect on subjective analysis of insomnia [35]. These findings are major for AUD endorsement as the inability to get a good night of sleep is a common complaint of AU and AUD, that contributes in some to the build‐up of stress, which leads to drinking episodes [24], and because sleep problems persistence during alcohol recovery increases relapse risk [2, 6, 36]. On the contrary, effective insomnia treatment after withdrawal increases abstinence [4]. Therefore, treating sleep problems of AUD could aid recovery and decrease relapse rate [6, 37].

Our findings also suggest that PA enhances subjective sleep duration and quality in AUD [26, 30] and in AU [32] [34], supported by a previously cited meta‐analysis, which also indicates that PA increases PSQI score—a questionnaire measuring sleep quality—and subjective TST as well as SE, SOL and WASO [35].

Thus, 80% of the studies from our review using subjective sleep analysis, supported by the literature, indicate that PA has a positive impact on sleep perception in AU and patients with AUD. Still, two studies based on AUD, also performing subjective sleep analysis, did not show significant enhancement following PA program. However, one of them is based on SUD patients, of which only two have AUD. This heterogeneous population, associated to the presence of PA in UC group, may explain the fact that insomnia reduction was identified however not significant [27]. As for the second one, it analysed sleep only through a 9‐point scale on ‘satisfaction with sleep’, not with scientifically validated questionnaire [25].

This review also highlights that PA induces long‐term (1 year) positive effect on subjective sleep quality among AU [34]. These results are consistent with 2023 review, indicating that regular exercise is a key factor in achieving long‐term sleep improvements in patients with sleep disorders, e.g., circadian rhythm disorder or insomnia, frequently diagnosed in AUD population [38].

#### 4.2.2 Sleep Outcomes Objectively Measured

Unfortunately, only one study used objective sleep analysis (7‐day actigraphy) and it was based on AU; thus, no conclusion on PA's impact on objective sleep of AUD patients can be done. Still, it showed that longer PA in the day reduces objective sleep fragmentation that night, for AU with lower consumptions than average (M = 3.96 units/week) [31]. These positive effects in AU are supported by a 2024 meta‐analysis showing that PA plays an important role in reducing WASO and improving SE objectively measured [35]. This is particularly interesting since alcohol misuse leads to increased WASO, sleep fragmentation [2] and poorer SE [2]. Thus, PA appears as a key factor in preventing alcohol‐induced sleep disorders in nonclinical population. However, individuals with higher consumption (above 3.96 units/week) did not receive the benefit described earlier in AU [31]. This could mean that alcohol misuse moderates the positive impact of PA on objective sleep parameters. Nevertheless, no study based on AUD measured PA's impact on objective sleep outcomes, which should be investigated judging by these findings to draw clear conclusion.

Results from our review, supported by recent meta‐analysis, precise PA's positive influence on subjective sleep outcomes of alcohol users with and without dependance and on objective sleep outcomes of AU without dependence. It also highlights the need for researchers to combine subjective to objective analysis of sleep in AUD. Using polysomnography to enable the measure of sleep structure variation would also be interesting as alcohol‐induced sleep stage variations [2, 4] are opposite to PA's impact on it, in non‐clinical population [15]. Thus, it would be interesting to know if acute and/or regular PA could reverse the negative impact of alcohol on sleep structure in AUD with active consumers, recently withdrawn patients and long‐term abstinent.

#### 4.2.3 Effect of Demographics

It should be noted that the selected studies focus on adults with ages ranging from 31 to 54.3 years in AUD and 26.7 to 42 years in AU. Regarding gender, women are underrepresented, especially in AUD with women's representation ranging from 0% to 30.4% against 18.4% to 61.4% in AU. No significant differences related to age or gender between studies arose. Yet, with those samples, conclusions of the present study cannot be generalized to younger or older population, especially since aging also impacts sleep. Furthermore, results should be taken with caution regarding women, especially in AUD, as they were clearly underrepresented.

### 4.3 Optimal Program

A final key point is to determine the optimal PA program to enhance sleep in AUD patients. Regrettably, although most of the studies show that PA has positive effects on sleep in AU and AUD, there is no general agreement on optimal program characteristics nor precautions regarding statute of treatment for AUD patients. Nevertheless, some conditions can be highlighted.

#### 4.3.1 PA Characteristics to Enhance Sleep Outcomes

Regarding *duration and intensity*, a study from this review, based on abstinent AUD, showed no significant enhancement on sleep outcomes following a 6‐week PA program including 10‐min sessions of yoga 5 days a week, and 5‐min jogging per day [25]. This tends to indicate that in withdrawn AUD, PA enhances sleep only if duration and intensity are sufficient. Indeed, a 2023 meta‐analysis found that especially high‐intensity exercise alleviates withdrawal symptoms in withdrawn SUDs, including AUD [39]. However, no study on withdrawn AUD measuring duration or intensity of PA's impact on sleep outcomes could be found to support this hypothesis.

All our studies were analysing *regular exercise* impact, in AU and AUD, and most indicated positive sleep outcomes, which is supported by previous studies showing that regular exercise can reverse alcohol‐induced brain impairments and cognitive dysfunction [40].

Studies from this review do not show significant differences in sleep outcomes related to *type of activity* as yoga, endurance or strength trainings came to similar conclusion, for either population (AU or AUD). Indeed, moderate and light intensity strength, mind–body exercise and moderate aerobic exercise can reduce the severity of insomnia and improve sleep quality [35].

Even though it is not mentioned in any of the selected studies, time of day seems to influence PA's effect on sleep. Indeed, morning exercise appears as the most effective since exercise practiced at least 6 to 8 h before bedtime [41] or at 7 AM has better impact on sleep than at 1 or 7 PM [17]. Yet, this has only been measured in nonclinical population; thus, it should be analysed in AUD to enable generalization of the results.

#### 4.3.2 APA Prescription and Precautions for AUD

With the evidence supporting the extensive benefits of exercise for AUD and sleep disturbances rapidly growing, the demand for clinical exercise interventions in AUD services is expanding. However, at present, there are no clear safety considerations or guidelines specific for APA in AUD. Given this lack, there is a call for developing standardized safety protocols. These guidelines would assist healthcare professionals in safely incorporating APA into treatment plans, considering factors like comorbidities and the patient's current alcohol use status (intoxication, withdrawal and abstinence). To enable the publication of guidelines, studies investigating the impact of several types of APA programs at different stages of the treatment (intoxication, withdrawal and abstinence) of AUD patient are needed.

Exercise should be endorsed by qualified professionals formed to APA in AUD population to reduce the risk of injuries, adapt activity to the patient status and potentiate benefits on health factors [42]. Besides, the presence of qualified trainers also enhances adhesion [43].

Although, given the wide variety of PA modalities presented inducing similar positive sleep outcomes in AU and AUD, participants could benefit from individually tailored exercise interventions based on their preferences and needs [18].

### 4.4 Adhesion

In AUD studies, offering PA program, 75% to 77.7% [26, 27] adherence rate was indicated and 55.55% dropout appeared [24]. These results show that even though most AUD patients have a sedentary lifestyle and attendance issues [44], they remain interested in PA, which is often preferred to pharmacological treatments. Indeed, a recent study showed that only 21% of AUD patients have high adherence to their medication [45]. Hence, PA program appears to be at least three times more attractive compared to standard medication. Finally, one study from this review specified adherence rate between groups, showing greater adhesion in exercise group, 75% against 58.3% in UC [27]. Nevertheless, AUD patients with insomnia are less likely to engage in PA (21% vs. 70%) [29], which could explain the lack of studies analysing the impact of PA on sleep in this population. Still, AUD patients with insomnia report higher dependence [29], thus, even though there might be adhesion difficulties, research is all the more necessary.

PA's attractiveness could be explained by its efficiency as it enhances not only sleep but also alcohol intakes [46], cravings [47] and abstinence [48]. It is also less stigmatising and has only few side effects compared to psychiatric pharmacological treatments. Furthermore, nonpharmaceutic treatment limits the risk of addiction transfer.

### 4.5 Limitations

Firstly, there most certainly is a lack of publication that led to the inclusion of only few studies with various study designs. Various samples also had to be included assessing AU, AUD and SUD. Besides, only few were RCT, and a high risk of bias remained in the ones included. Researchers should now work on well‐designed RCT to analyse the matter and provide robust results.

Assessing the impact of PA on sleep in AU was not the main purpose of any of the studies included. Thus, alcohol use and/or sleep and/or PA was usually not precisely described. This resulted in the presence of several limitations. First, given the dynamic nature of alcohol‐induced sleep changes, it would have been necessary to discuss alcohol history and compare the impact of PA on sleep in relation to current alcohol use status (intoxication, withdrawal and abstinence). However, we were not able to do so as we could not find precise information on alcohol history in 9 out of 11 studies. It also prevented analysing and discussing the timing of the PA program and sleep analysis in relation to withdrawal. Should be added that only one study used objective sleep analysis, another limitation. The presence of treatments for AUD or aimed at improving sleep could also not be discussed as we could not find precise information, while it could interfere with PA's impact on sleep. In that regard, we suggest to upcoming studies to present precisely factors influencing sleep such as alcohol history and concurrent medication.

Hence, this review's results are to be treated with caution in regard to several limitations. Nevertheless, it still highlights promising results regarding benefits of PA on sleep in alcohol users with or without AUD, and even more so, the importance of performing new well‐designed research on the question.

## 5 Conclusion

Out of 4995 articles screened, none assess as primary purpose the impact of PA on sleep in AU or AUD. Nevertheless, 81.8% of the selected studies, in their secondary outcomes, highlight PA's positive association with sleep in alcohol users with or without dependence. Thus, APA should be envisioned as a suitable therapeutic in the treatment of AUD's sleep disturbances, which is crucial given that sleep problems persistence during alcohol recovery increase relapse risk. These encouraging results highlight the need for new well designed research assessing sleep with objective and subjective tools and offering well‐designed PA program on representative samples. Furthermore, since adhesion in this population appears as an impediment for research, feasibility study could be recommended. Another step would be to compare several PA programs, on AUD patients with various dependence statuses, to create tailored program enhancing sleep outcomes and precise guidelines on how to endorse APA with AUD patients.

## Author Contributions

PhD student Lilou Duquet had the original article idea, screened articles and wrote the systematic review. Dr Julie Giustiniani, psychiatrist specialising in addictology, co‐screened articles, supervised the writing and reviewed the manuscript. Dr Silvio Galli, neurologist specialising in sleep, reviewed and corrected the manuscript concerning sleep outcomes. Professor Emmanuel Haffen, psychiatrist, ensured resources needed were available and directed PhD student and first author's work.

## Conflicts of Interest

The authors declare no conflicts of interest.

## Data Availability

Data sharing not applicable to this article as no datasets were generated or analysed during the current study.
